# Exploring the Public Health of Travel Behaviors in High-Speed Railway Environment during the COVID-19 Pandemic from the Perspective of Trip Chain: A Case Study of Beijing–Tianjin–Hebei Urban Agglomeration, China

**DOI:** 10.3390/ijerph20021416

**Published:** 2023-01-12

**Authors:** Shuai Yu, Bin Li, Dongmei Liu

**Affiliations:** 1 Research Institute of Highway Ministry of Transport, Beijing 100088, China; 2 National Intelligent Transport Systems Center of Engineering and Technology, Beijing 100088, China; 3 Research and Development Center of Transport Industry of Big Data Processing Technologies, Beijing 100088, China

**Keywords:** travel behavior, trip chain, COVID-19, intercity travel, urban agglomeration, public health

## Abstract

The outbreak and spreading of COVID-19 since early 2020 have dramatically impacted public health and the travel environment. However, most of the studies are devoted to travel behavior from the macro perspective. Meanwhile, few researchers pay attention to intercity travel behavior. Thus, this study explores the changes in the travel behavior of intercity high-speed railway travelers during the COVID-19 pandemic from the perspective of the individual. Using the smartphone data, this study first extracts the trip chains by proposing a novel method including three steps. The trip chain can describe the whole process of traveling, including individual characteristics, travel time, travel distance, travel mode, etc. Then, a Multinomial Logit model is applied to analyze the trip chains which verified the validity by using studentized residual error. The study finds that intercity travel behavior has changed in gender, age, travel mode choice, and travel purpose by comparing the trip chains between May 2019 and May 2021 in the Beijing–Tianjin–Hebei urban agglomeration. The method proposed in this study can be used to assess the impact of any long-term emergency on individual travel behavior. The findings proposed in this study are expected to guide public health management and travel environment improvement under the situation of normalized COVID-19 prevention and safety control.

## 1. Introduction

A series of infectious diseases such as Severe Acute Respiratory Syndrome (SARS), Middle East Respiratory Syndrome Coronavirus (MERS), Ebola, etc., have seriously threatened people ranging from public health to psychological well-being. These contagions can also revolutionize people’s lives in other aspects, for example through huge economic losses, travel environment, and education or career interruption [1,2]. However, the COVID-19 pandemic, which broke out at the end of 2019 and quickly expanded at the beginning of 2020, has had more continuous and extensive influence on the entire mankind. By 14 November 2022, 631,935,687 cases and 6,588,850 deaths had been confirmed across the world [3]. Contemporarily, COVID-19 is an enormous social risk that has formed tremendous threats to the world [4] on various aspects of society, including public health, environmental systems [5], aviation [6], agriculture [7], foreign trade [8], political relations [9], etc., ranging from people’s mental health to social stability [10]. Moreover, because the pandemic is very contagious and spreads through close contact with infected persons, COVID-19 has had a tremendous and unprecedented impact on daily life’s behaviors, i.e., hosting, transportation, traveling, communication, etc. and the extent of the changes in travel behavior wrought during and after the pandemic remains unclear.

In order to protect public health, a range of policies and measures have been proposed to control the virus around the world, such as social distancing, school closures and quarantining, severely restricting travel, and many other activities globally. Considering the human-to-human nature of COVID-19 transmission, urban lockdowns and travel restrictions are major policies to counter its spread. As the first country to discover coronavirus, China has launched a first-level emergency response to this public health emergency and rigidly enforced nonpharmaceutical interventions (NPIs) to constrain COVID-19 from 19 January 2020, to the present, including formulating a general strategy of “preventing the coronavirus from re-entering the country to cause a new epidemic” and the dynamic zero-COVID policy [11,12]. Therefore, China has implanted a variety of more strict measures to make every possible effort to curb the spread of the virus, such as lockdowns, travel restrictions, and wearing masks in public spaces.

Because of the strict NPIs, in China, the pandemic is now generally under control and the transport environment has returned to nearly normal. There have been 8,771,347 confirmed cases and 29,370 deaths reported, respectively 1.4% and 0.4% of the world level, while the population of China is nearly 17.7% of the global population by 14 November 2022 [13]. Currently, the continually mutating COVID-19 virus has induced a long-term period of recovery, i.e., the post-COVID-19 phase, which refers to the period of relative and regional stability after the initial uncontrolled global outbreak and growth of the pandemic, which has been referred to as the “new normal” by the WHO [14]. Although the COVID-19 pandemic was well-controlled to some extent, these unprecedented measures have had a profound impact on the number and purposes of trips and modes of travel for a long time [15]. Traffic flow has been restored gradually in different places, however, people’s travel behavior is still affected by traffic control measures to different degrees.

Intercity travel has more risks than traveling within a city in virus transmission because intercity travel increases the scope of people’s communication to some extent [16,17]. The roles of High-Speed Rail (HSR), aviation, and coach in the spread of COVID-19 have been investigated in China, and the presence of the HSR hub was significantly related to the pandemic diffusion [18]. By 2019, more than 224 inland cities in China were connected by HSR, and the total mileage of HSR had reached 35,000 km, according to China’s “Mid-to-Long-Term Railway Network Plan (Revised in 2016)” [19]. With the development of HSR, the travel time between cities has been shortened, and commuting between urban agglomerations has gradually increased. Exploring the changes in the travel behavior of intercity High-speed railway travelers during the COVID-19 pandemic is very important for transportation policy formulation and sustainable development in the post-COVID-19 phase and normal daily context.

In terms of COVID-19 impacts, some of the knowledge about travel behavior has been accumulated from early academic reviews, reflections, and visions that were based on theoretical reasoning or investigations of future traveler behavioral intentions [20]. Significant efforts have been devoted to investigating the impact of the COVID-19 pandemic on tourist arrivals or changes in travel behavior [21,22,23]. Most empirical studies pay attention to changes in travel frequency and mode choice, while few researchers focus on traveler characteristics and travel structure, both before and after the emergence of COVID-19 [24]. Meanwhile, massive objective traffic-related data are generally owned by governments and big corporations, and most scholars use questionnaires to explore the change in travel behavior, which limits the researchers to quantify the changes in individual behavior under the impact of COVID-19.

Nowadays, with traveler behavior changes and the related effects resulting from the pandemic possibly being long-lasting, it is important to analyze the current situation and discuss the impacts of COVID-19 from the perspective of different individuals and groups. This study aims to explore the relationship between the impacts of the COVID-19 pandemic and changes in travel behavior in urban agglomeration from the perspective of individuals’ whole travel procession, i.e., trip chain. Based on smartphone data which costs less and contains more information, this study proposes a novel method to identify intercity travelers using HSR and generate trip chains within Beijing–Tianjin–Hebei urban agglomeration. Then, a multinomial logit model is employed to reveal the changes in travel behavior by analyzing the intercity trip chains. To increase the persuasiveness of the results, smartphone data from the same month (May) in different years, which are the period before the outbreak of the pandemic and the post-COVID-19 phase, respectively, is used in this study. Understanding residents’ trip chains provides critical support for various applications in public health, the transportation environment, and many other related fields. The results of this study can support public health management and travel environment improvement [25,26].

The rest of the paper is organized as follows: in the next section, a review is provided of the relevant literature. Section 3 presents an explanation of data processing. Section 4 describes the methodology. The empirical results are subsequently presented and discussed in Section 5. Finally, conclusions and recommendations for future works are outlined in Section 6.

## 2. Literature Review

### 2.1. Impact of COVID-19 on Travel Behavior

A number of researchers have analyzed the impact of COVID-19 on the transportation system, such as the pandemic spread through transportation [27,28], public transportation [29,30], travel demand [31,32], travel mode choice [33,34], etc., after the outbreak of the pandemic, which indicates that people’s travel behavior has changed under the threats of the virus and the related strict restrictions. Recognizing the underlying links between travel behavior and public health emergencies, researchers attempted to analyze the changes in travel behavior caused by COVID-19 [35]. Notably, changes in travel behavior are generally studied from the perspective of macroscopic and individual.

A great part of the studies explored the changes in travel behavior from the macroscopic perspective using open statistics. Some researchers analyzed the impact of COVID-19 on population migration and urban traffic and found that while implementing anti-pandemic measures, the relevant government departments should concentrate more on densely inhabited and economically developed provinces and cities [36]. Based on Baidu migration data, some researchers adopted a cluster analysis of origin and destination to analyze the relationship between migration and pandemic progression in the Guangdong-Hong Kong-Macao Greater Bay Area [37]. It is believed that the degree of pandemic progression in the Guangdong-Hong Kong-Macao Greater Bay Area is positively correlated with the degree of immigration from Wuhan to the Greater Bay Area. The performance of the transportation system has been impeded to some extent under the influence of COVID-19 and the relevant policies. Although the transportation system has progressively recovered during the post-COVID-19 period [38], the operation of the transportation system is positively related to the spread of the pandemic [39]. Due to the pandemic, in Spain, travel volume dropped dramatically while public transportation is extremely affected and private cars are relatively less affected [40]. The amount of travel volume in the State of Qatar decreased by around 30% after the travel restrictions, while the distribution of all-day travel did not change significantly [41]. Travel volume and distance have also obviously decreased in other countries, with about 55% of travel volume and 68% of distances traveled in the Netherlands, 90% of travel volume using public transportation, and 60% in daily distance traveled [42]. This type of study mainly pays attention to urban transportation systems employing various types of data to withdraw indicators to describe the characteristics of the urban transportation system, such as traffic volume, traffic structure, travel frequency, and travel mode choice [43,44].

A series of studies focuses on analyzing and simulating the changes in travel behavior from the perspective of the individual. Travel behavior of individuals is affected by differences in the gender, education level, lifestyle, travel purpose, etc., of travelers [45,46]. An online survey was employed in Tokyo, Japan, to explore the participation of urban residents in traveling during the early stage of COVID-19 [47]. It was found that people have voluntarily avoided leisure activities and dining outside since the early stage of the pandemic. The phenomenon of online activities such as online learning, working, shopping, etc., were adopted, and the change in transportation mode to walking and cycling are revealed through a preference-stated preference (RP-SP) survey in Chicago, USA, during the pandemic [48]. The number of travelers who aimed at education, visiting friends, and personal care decreased the most among all the purposes of travel in the Netherlands [49]. The frequency of travel for social, economic, and religious activities, in Nigeria, has considerably decreased based on a combination of data from active questionnaire surveys via email, social media, and professional networks [50]. In several Chinese cities, i.e., Beijing, Shanghai, Guangzhou, Shenzhen, and Chongqing, the frequency of travel for shopping, tourism, eating out, and taking public transportation during the peak of the COVID-19 was significantly reduced, as compared to the period of outbreaking of the pandemic. Based on the license plate recognition data, the adjustment of travelers’ behaviors under the influence of the pandemic was analyzed in Yiwu, Zhejiang Province, China [51]. In China’s Greater Bay Area, it was found that, in comparison to advantaged groups, socially disadvantaged groups experienced a steeper decline in travel mobility during the pandemic’s peak, but a more significant recovery afterward by analyzing mobile phone data [52]. This type of study mainly attempted to describe the changes in travel behavior from the individual perspective through questionnaire data, mobile phone data, and so on.

In order to analyze the changes in travel behavior more comprehensively and accurately, this study explores the changes in the travel behavior of intercity high-speed railway travelers during the COVID-19 pandemic from the perspective of the individual. Additionally, a novel method to generate trip chains of intercity High-speed railway travelers, which includes identification of intercity travelers, extraction of travel features within the city, and generation of intercity trip chains, is first proposed in this study.

### 2.2. Smartphone-Based Trip Chain

For decades, numerous researchers have explored the character of people’s travel behavior, which is measured as daily trip frequency, trip purposes, departure time, travel duration, travel distance, travel modes, trip sequences, trip destinations, travel companions, etc., which can be displayed in trip chains, to provide long-term guidance and short-term strategies for urban planning and transportation development [53,54,55,56,57,58,59,60,61,62]. A trip chain is a collection of interconnected trips that begin at one origin, pass through one or more stops, and then end at a destination. It was first used to test the influence of pre-planning activities on a certain day in the trip chain on the traffic pattern of a day from the perspective of utility maximization in 1979 [63].

Traditionally, data used in trip chains were mainly obtained from travel surveys. Travel surveys are important data for analyzing and evaluating travel behavior daily. A typical household travel survey is meant to collect extensive information about travel and activity behavior from a sample of houses or people. Nevertheless, a city-wide household travel survey is often costly to collect, lacks instantaneity and continuity, short survey duration, has incomplete information, and quickly becomes out-of-date [64,65,66,67]. 

With the development of technology, Global Positioning System (GPS) is used in travel surveys. The first use of GPS in a travel survey was in Austin, Texas, in 1996, when 200 families were requested to describe their trip chains through computer-assisted telephone interviews while concurrently employing GPS in their vehicles to track vehicular trips [68]. Due to vehicle GPS loggers only collecting vehicular trips and having technical issues, such as a cold start signal acquisition delay, there are obvious limitations for vehicle GPS loggers to collect travel by all modes [69]. With the use of wearable GPS devices, travel surveys are getting more attention because they can collect higher-quality data by continually and correctly capturing respondents’ travel paths without introducing hassles to respondents [70]. However, limitations still exist which restrict GPS use in travel surveys, for example, the price of GPS devices is expensive and the fully productive response of GPS devices is low.

Recently, with the advancement of information and communication technologies, many kinds of novel data, like smartphone data, have been used to trip chain research. Smartphone data can not only capture the real-time location information of travelers, but also describe the attributes of individuals, such as age, gender, destination type, and so on. Compared with traditional survey data and GPS data, smartphone data have several clear advantages, such as lower costs, more locational sensor data sources, more accurate data, wider geographic area, more coverage of population, and greater likelihood for respondents to be collecting data at all times throughout the survey period, which attract scholars in various fields to apply them to travel behavior research, and a certain amount of progress has been made to date [71]. Some studies employed smartphones to passively collect GPS data with minimum user interaction or input on the app [72], while few researchers make efforts on extracting trip chains from smartphone data. In order to overcome the above limitations, this study generated intercity trip chains with smartphone data which are from May of 2019 and 2021.

## 3. Data Description and Processing 

### 3.1. Study Area

**Figure 1:**
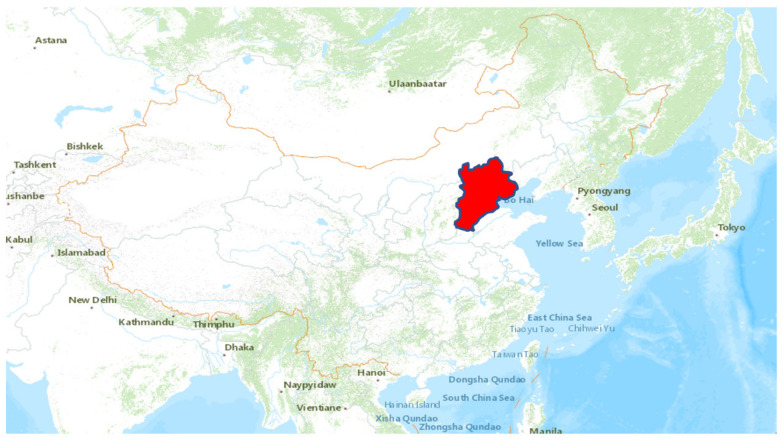
Study area: Beijing-Tianjin-Hebei urban agglomeration.

In this study, the travel behavior of travelers who use the high-speed railway for intercity travel between Beijing, Tianjin, Shijiazhuang, and Handan, within the Beijing–Tianjin–Hebei urban agglomeration, as shown in Figure 1, is investigated. Beijing–Tianjin–Hebei urban agglomeration, which is a capital economic circle and the center of the political and cultural center, plays an important role in the development of China. It is also an essential part of the Chinese transportation system because it is one of the four important international terminal clusters in the guidelines of China’s comprehensive national transport network. Beijing, Tianjin, Shijiazhuang, and Handan are the core cities in the Beijing–Tianjin–Hebei urban agglomeration which have the largest and most representative inter-city traffic volume in the urban agglomeration. The data period of this study is May 2019 and May 2021, which are before the outbreak of COVID-19 and the period of the post-COVID-19 phase, respectively. In China, May includes a five days’ vacation which is the Labor Day holiday and does not includes long vacation, such as winter vacation and summer vacation. In addition, the climate in May is relatively comfortable for traveling. Therefore, the data in May is representative to some extent. Since April 2020, Chinese residents resumed travel, and the tourism industry was gradually recovering [73]. China has entered into a special and unique recovering period that is distinct from the other countries that were still undergoing serious impact from COVID-19.

### 3.2. Smartphone Data

The raw data used in this study is smartphone data, and it is collected from the platform of Data-as-a-Service (DAAS) which is operated by one of the Chinese communication operators with a 30% market share within the Beijing–Tianjin–Hebei urban agglomeration. The raw data has several kinds of information as follows. The first type of data is basic location data which includes the location information of mobile communication base stations, the grid where the base stations are located, the road node, the latitude and longitude coordinates of the base stations, etc. The second type of data is residence data. According to the system setting, a user’s stay point is supposed to be a location where he stays for more than 30 min, which includes the start and end time of presence, location, frequency of presence, etc. The location data in this study follow the principle of World Geodetic System 1984 (WGS84). The third type of data is travel data which includes travel start and end time, travel location, travel speed, travel time, travel through base stations and road nodes, etc. The fourth type of data is user attribute data which stores basic personal information, internet service records, and other related information. The fifth type of data is coding data which stores the user’s ID card domicile location, internet access behavior label, location, and other information. This study collects a grand total of 27,120,000 attribute information of smartphone users and 54,900,000,000 travel data, as shown in Table 1.

### 3.3. Data Processing

Due to the generation mechanism of smartphone data and the data collection system, there will be invalid data and noise data such as duplicate, missing, and wrong data in the raw data, which will generate an adverse effect on extracting trip chains. First, a clustering algorithm of k-means was used to clean the noise data, which were erroneous or abnormal, such as unknown gender, unknown age, etc. [74] The software of Statistical Package for the Social Sciences (SPSS) was used to complete the k-means algorithm. The raw data was imported to SPSS in step 1. The method of iteration and classification were chosen in step 2. The number of iterations were set by 10, while the convergence conditions default to 0 in step 3. Then, the k-means algorithm was completed and the results were showen in SPSS. The noise data which were erroneous or abnormal were cleaned. Secondly, in order to facilitate the analysis of results, this study discretizes the variables of age, distance and time as shown in Table 2. 

Lastly, a series of variables, such as road number, smartphone brand, internet traffic, etc., which are not related to generating trip chains are removed, while a total of 15 variables are selected as shown in Table 3.

## 4. Methodology

### 4.1. Trip Chain Generation

**Figure 2:**
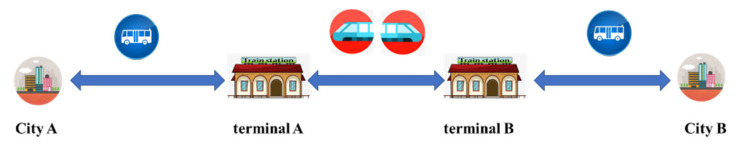
Schematic diagram of urban agglomeration trip chain.

The trip chain of urban agglomeration is not only a simple process for individuals to reach the ending point from the starting point. It also contains a lot of information, such as travel time, space trajectory, mode of transportation, and so on, which are interrelated and interact with each other and are accompanied by the whole process of travel. Travel purpose and mode choice are simultaneously decided by travelers [75,76]. In this study, a trip chain refers to a whole process of intercity travel which includes travel within the origin city, intercity travel by using a high-speed railway, and travel within the destination city as shown in Figure 2. The method to generate trip chains is as follows.

#### 4.1.1. Identification of Intercity Traveler

In this study, as the data of the DAAS platform shows, the user stay data is extracted intelligently in the unit of the city. Therefore, in order to identify intercity travelers, we use the following method:

Step 1: Generate a hub layer based on the geographic location of the research hub, convert it into a wkt file, and import it into the DAAS platform;

Step 2: Spatial matching, if the user stays in the space range of the research hub, then extract the user’s data as the hub-staying user data set;

Step 3: Data grouping: group the data set according to users, hubs, and dates to obtain the time series of each user’s stay in each research hub every day;

Step 4: Travel generation: extract the user’s last stop at the current hub and the first stop at the next hub, the travel between the two stops is the user’s intercity travel between the hubs.

**Figure 3:**
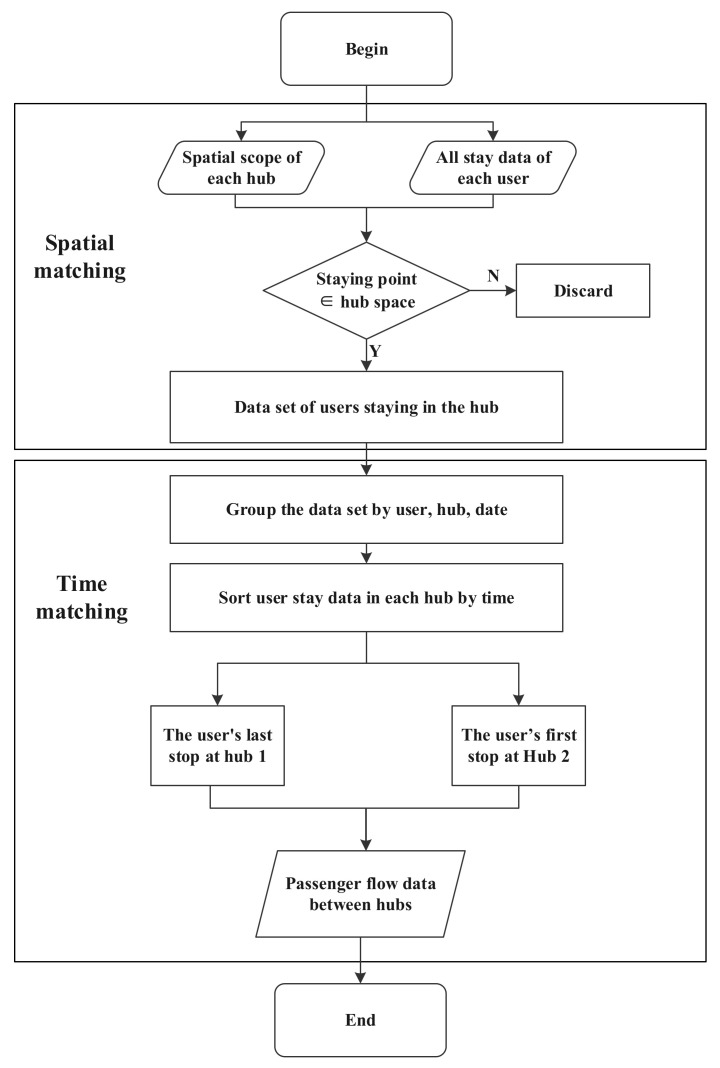
Extraction of intercity travel OD.

The technical route of this part is as shown in Figure 3:

#### 4.1.2. Extraction of Travel Feature within the City

This study uses a two-layer clustering algorithm based on time and space to identify the user’s travel stop point. The algorithm is divided into two stages.

In the first stage, we use the Density-Based Spatial Clustering of Applications with Noise (DBSCAN) algorithm to cluster user trajectory data spatially. Then, we perform time clustering on the result data of the first stage, and finally, accurately identify the user’s staying point, and then obtain the user’s travel OD. The specific steps of this algorithm are as follows:

Step 1: Extract all trajectory data of relevant users in the research area;

Step 2: Through all the trajectory data of each user and input them as data;

Step 3: Determine the parameters of the DBSCAN algorithm, including the minimum number of stay points (MinP) and the minimum distance of stay points (MinR);

Step 4: The DBSCAN model calculates and outputs the stay points of each user;

Step 5: Sort the stay points in time series, and calculate the stay time of each cluster;

Step 6: Determine whether the stay time of each cluster is greater than the time threshold T. If it is not, it indicates that the residence time is not matched to the stay point;

Step 7: Output a set of stay points based on the identified stay points and connect them to form the travel OD of each user.

**Figure 4:**
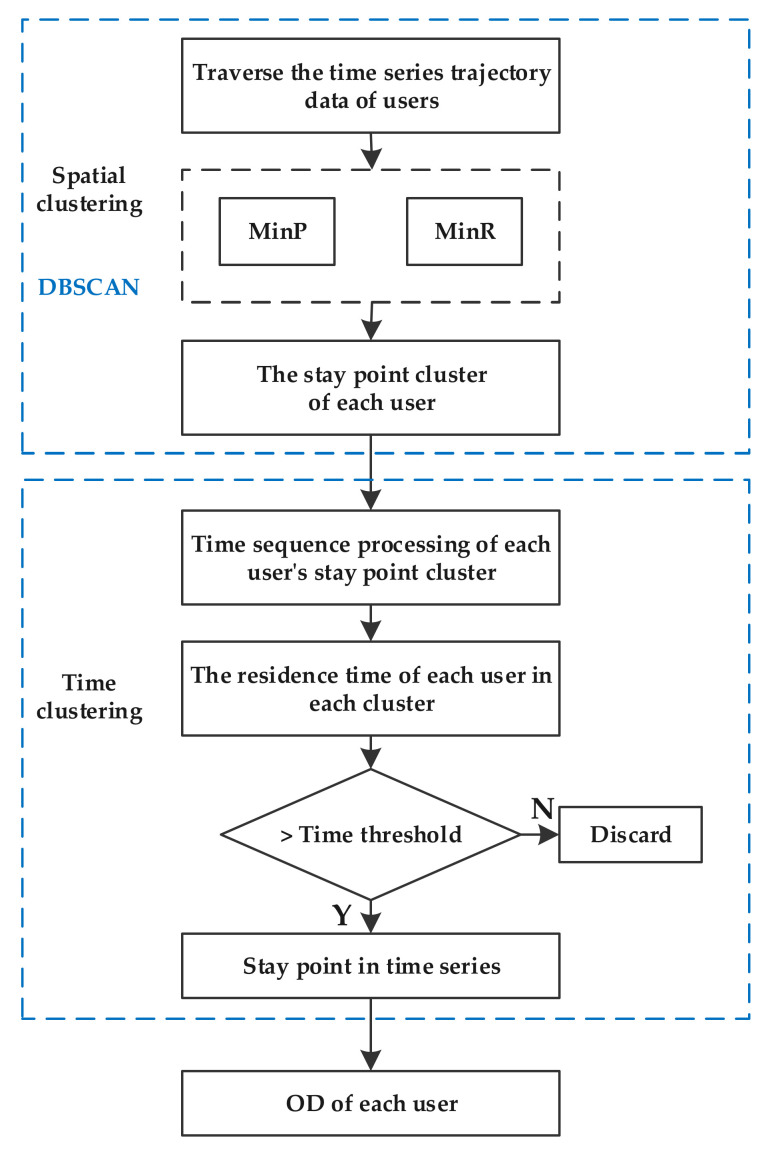
Travel OD extraction algorithm based on spatio-temporal clustering.

The technical route of this part is as shown in Figure 4:

In this algorithm, it is necessary to adjust and verify the MinP and the MinR in the DBSCAN algorithm, and T in time clustering, or set it based on experience. In this study, we determined the MinP as 8 according to the average number of daily trajectory points of the user, and then we compared the results of identification under different MinR and T. According to the 5th Beijing Comprehensive Transportation Survey (2014), the average trip frequency of the residents is 2.75. We input different threshold values into our OD algorithm and then compare the calculated results with the above survey data. It can be found from Table 4 that the trip frequency is closest to the travel survey result when T is set as 30 min and MinR is set as 500 m. Additionally, this also indicates that the result of our OD algorithm is consistent with the actual survey data, which proves the accuracy of the algorithm.

#### 4.1.3. Generation of Intercity Trip Chains

Travelers may have several trips within origin city and destination city, but we only need the trips that include hub A and hub B. Therefore, after extracting the OD of each user’s intercity travel and the user’s intercity travel OD between the research hubs, we match the user’s entire trip chains between two cities by filtering the trips which included hub A and hub B in the same day. The rules are as follows:

Step 1: The same user on the same day:

Step 2: The user’s travel destination of the current trip in city A is the research hub;

Step 3: The user’s next trip will be an intercity trip, and the destination of the trip will be the research hub of city B;

Step 4: The user takes the research hub of city B as the starting point to generate trips in city B.

**Figure 5:**
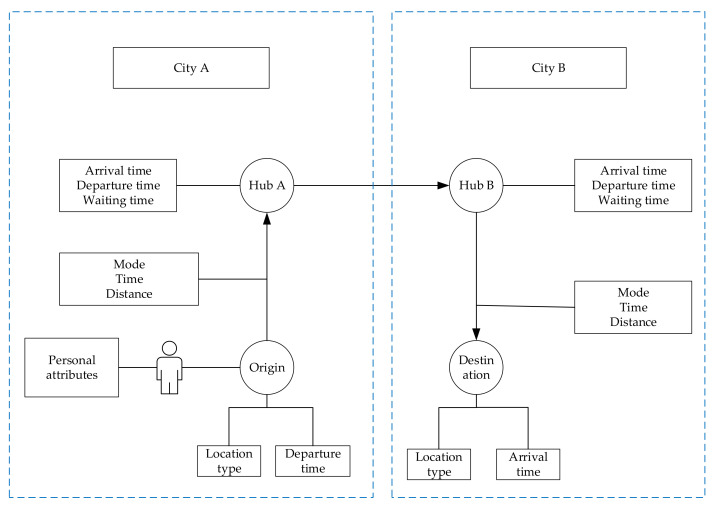
The process of generating individual trip trains.

The process of generating individual trip trains is shown in Figure 5:

### 4.2. Validity Test of Trip Chains

The data used in this study has the characteristics of large size, high dimension, and discretization. Therefore, the existing mainstream outlier detection methods such as simple box plots cannot do anomaly detection for categorical data [77,78,79,80]. Under the assumption that travel time increases with travel distance, the two variables of travel distance and travel time (in fact, a total of four variables, divided into the departure process and arrival process) can be jointly detected (multivariate outlier detection, the joint interaction of multiple variables to detect anomalous data records.). Therefore, the studentized residual error of least squares linear regression model is used to identify abnormal values. The mathematical formulation can be characterized as follows:

(1) $$y=\beta_{0}+\beta_{1}x+e$$

Here, the exploratory variable *x* is travel distance while the explained variable *y* is travel time. $\beta_{0}$ is the intercept and $\beta_{1}$ is the gradient. e is the random error term which is set in linear regression model. The least square method is used to fit the model and estimate the parameters. The parameter estimation formula is as follows:

(2) $${\hat{\beta }}_{1}=\frac{\sum_{i=1}^{n}y_{i}x_{i}-\frac{\left(\sum_{i=1}^{n}y_{i}\right)\left(\sum_{i=1}^{n}x_{i}\right)}{n}}{\sum_{i=1}^{n}x_{i}^{2}-\frac{{\left(\sum_{i=1}^{n}x_{i}\right)}^{2}}{n}}$$

(3) $${\hat{\beta }}_{0}=\bar{y}-{\hat{\beta }}_{1}\bar{x}$$

Here, *x_i_* and *y_i_* are, respectively the explanatory variable and explained variable of the ith data. n represents the total number of the data. $\bar{y}$ is the arithmetic mean of the explained variable, while $\bar{x}$ is the arithmetic mean of the explanatory variable. $\hat{\beta }$ and ${\hat{\beta }}_{1}$ are, respectively the least squares estimate of $\beta_{0}$ and $\beta_{1}$. The fitting model is as follows:

(4) $$\hat{y}={\hat{\beta }}_{0}+{\hat{\beta }}_{1}x$$

The ordinary residual of each data is as follows:

(5) $$e_{i}=y_{i}-{\hat{y}}_{i}=y_{i}-\left({\hat{\beta }}_{0}+{\hat{\beta }}_{1}x_{i}\right)$$

Next, let $\;y_{i}\;$ denote the observed response of the *i^th^* observation in the dataset, and $\;{\hat{y}}_{i\_del}$ denote the predicted response of the ith observation using the model fitted to the dataset after the *i^th^* observation has been removed. The *i^th^* deleted residual is defined as:

(6) $$d_{i}=y_{i}-{\hat{y}}_{i\_del}$$

The studentized residual $\;t_{i}\;$ for each observation is then calculated by dividing its deletion residual by its estimated standard deviation:

(7) $$t_{i}=\frac{d_{i}}{s\left(d_{i}\right)}=\frac{e_{i}}{\sqrt{MSE_{\left(i\right)}\left(1-h_{ii}\right)}}\sim t\left(n-p-1\right)$$

This turns out to be equivalent to the ordinary residual divided by a factor that includes the mean square error based on the estimated model with the *i^th^* observation deleted, *MSE*(*i*) (refers to the mean square error of the fitted model after removing *i^th^* observation) and the leverage, $h_{ii}\;$(refers to the diagonal elements of the “hat matrix”, which corresponds to the *i^th^* observation). In addition, $t_{i}$ obeys a T-distribution with n−p−1  degrees of freedom and 95% confidence level, n  refers to the dataset size and  p  refers to the number of parameters of the fitted model.

### 4.3. Multinomial Logit Model

A trip chain is a record of the whole process of a traveler which includes age, gender, mode choice, travel purpose, travel distance, travel time, and so on. In this study, a multinomial logit regression model with trip chains as the dependent variable is constructed to explore changes in travel behavior. Firstly, a utility function is introduced as follows:

(8) $$T_{ij}=x_{i}\beta_{j}+\varepsilon_{ij}\left(i=1,2,\ldots ,n\right)$$

where *T_ij_* is the utility of individual *i* choosing trip chain *j*. *x_i_* is a set of individual attributes and travel characteristics while *β_j_* is the parameter vector to be estimated. *ε_ij_* denotes the error term. The probability of individual *i* choosing trip chain j is as follows:

(9) $$P_{i}\left(j\right)=P\left(T_{ij}>T_{im}\right),\left(m\neq j\right)$$

When *ε_ij_* follows the generalized extreme value distribution, the model formula is as follows:

(10) $$P_{i}\left(j\right)=\frac{exp\left(x_{i}\beta_{j}\right)}{\sum_{m=1}^{J}exp(x_{i}\beta_{m})}$$

## 5. Results and Discussion

### 5.1. Generation and Validity Test of Trip Chains

**Figure 6:**
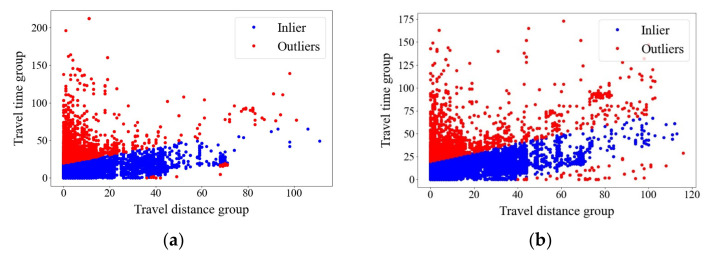
(**a**) Identification of abnormal value based on the variables of destination travel distance and time (May 2019); (**b**) Identification of abnormal value based on the variables of destination travel distance and time (May 2021).

Based on the dataset, the current study then used the mathematical programming software Python 3.5 on an Acer laptop with Intel Core i5–10210U 1.60 GHz 2.11 GHz CPU and 12 GB RAM to generate trip chains. There were 614,285 pieces of trip chains generated, which include 351,340 pieces of trip chains in May 2019 and 262,945 pieces of trip chains in May 2021. Next, the method of studentized residual was applied to test the validity of the trip chains. Firstly, the studentized residuals were calculated through simulating the model with the variables of destination travel distance and time, which was used to analyze the abnormal data points. The results are shown in Table 5 and Table 6, and Figure 6. In Table 5 and Table 6, the data whose travel distance from hub B to destination (DTD) are not match the travel time from hub B to destination (DTT) are deleted. For example, in the first data of Table 5, the value of DTD is “11” which equals 50–55 min, while the value of DTT is “212” which equals 1055–1060 km. In this situation, the travel speed is 1152–1271 km/h which is extremely abnormal. Figure 6 shows the results of identification of abnormal value based on the variables of destination travel distance and time. The red data points are qualified data while the blue data points are abnormal data.

**Figure 7:**
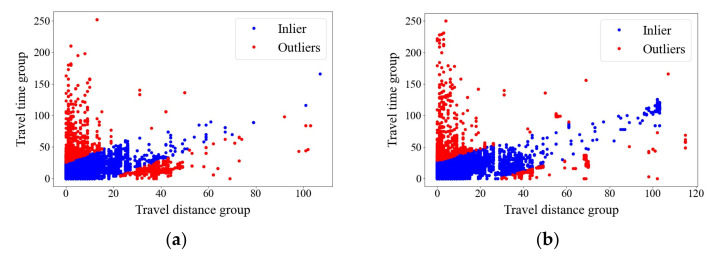
(**a**) Identification of abnormal value based on the variables of origin travel distance and time (May 2019); (**b**) Identification of abnormal value based on the variables of origin travel distance and time (May 2021).

Then, the anomalous value was deleted from the data set of trip chains. Based on the updated set of trip chains, the variables of origin travel distance, and time were used to detect the abnormal value. The results are shown in Table 7 and Table 8, and Figure 7. In Table 7 and Table 8, the data whose travel distance from origin to hub A (OTD) are not match the travel time from origin to hub A (OTT) are deleted. For example, in the first data of Table 7, the value of OTD is “13” which equals 60–65 min, while the value of OTT is “252” which equals 1255–1260 km. In this situation, the travel speed is 1167–1255 km/h which is extremely abnormal. Figure 7 shows the results of identification of abnormal value based on the variables of origin travel distance and time. The red data points are qualified data while the blue data points are abnormal data.

There was a total of 574,221 pieces of trip chain obtained after the validity test. The number of trip chains was 327,877 with removing 9950 trip chains in the origin process and 13,513 trip chains in the destination process, respectively from the data set of May 2019. The number of trip chains was 246,344 with removing 1545 trip chains in the origin process and 15,056 trip chains in the destination process, respectively, from the data set of May 2021.

### 5.2. Results of Multinomial Logit Model

The dependent variable of the multinomial logit model is Y which is the combination of travel mode. The independent variables of the model are shown in Table 9.

According to the input requirements of the stats model package, the independent variable Y, i.e., the combination of travel mode, was transformed to values 0, 0.2, 0.4, 0.6, 0.8, and 1 which, respectively represent private car–HSR–private car, private car–HSR–railway, private car–HSR–public transportation, railway–HSR–private car, public transportation–HSR–private car and public transportation–HSR–railway. The dependent variables were also transformed. Male was the reference of the variable of gender while the value of female was transformed to [T.2]. The remaining dependent variables were transformed as the same. Therefore, the results of the model are shown in Table 10, Table 11 and Table 12.

### 5.3. Discussion

#### 5.3.1. Analysis of Travel Behavior in May 2019 from Table 10

- From the perspective of gender, the coefficient of C (Gender) [T.2] is negative in all modes, which indicates that women are more likely to use the private car–HSR–private car trip chain for travel, followed by private car–HSR–public transportation. When Y equals 1, the coefficient of C(Gender) [T.2] is smallest in all modes which indicates that women refuse to travel by public transportation–HSR–private car a lot. The reason is probably that women usually travel more from developing cities to developed cities.
- Weekdays have a positive effect on the division of the travel mode combination of private car–HSR–railway and private car–HSR–public transportation, which indicates that travelers are more likely to arrive at the hub by private car. It is because more business trips are generated on weekdays and travelers tend to choose comfortable travel modes, while travelers prefer economic travel mode on weekends.
- Most age groups tend to travel through private car–HSR–railway. Moreover, travelers whose ages are from 19 to 39 are also likely to choose to travel between intercity without a private car, and middle-aged people travel more often compared to other age groups [81,82]. Individuals whose age is over 60 do not choose public transportation to travel within the intercity, which is opposite within the city.
- Travel distance has a significant in travel mode choice. Travelers prefer to travel by public transportation when travel distance is short.
- Considering the waiting time at the starting city transportation hub, overall, as the waiting time increases, the willingness of the traveler to choose this trip chain starts decreasing. A comparison between trip chains shows that travelers arriving at a transportation hub by railway are more likely to accept longer waiting times.
- The probability of choosing a railway (Y = 0.2, Y = 1) is higher than choosing other travel modes in the stage of traveling within the arrival city with the increasing waiting time, which indicates that there probably are problems in the process of transfer at the destination hub.
- Intercity travelers who travel from home have a significant positive effect on the travel modes of the private car by analyzing the coefficient of C (Activity_type)[T.10] and C (Activity_type)[T.12]. Intercity traveling to visit or work increases the probability of choosing public transportation.
- According to the constant term, the travel mode combinations of private car–HSR–private car and private car–HSR–public transportation are more popular than other combinations when the other conditions are the same.

#### 5.3.2. Analysis of Travel Behavior in May 2021 from Table 11

- The intercity travel behavior of women has changed dramatically under the impact of COVID-19. The number of male and female intercity travelers is 213,346 and 137,994, respectively, accounting for 60.7% and 39.9% of the total number of intercity travelers, respectively in May 2019, while the number of male and female intercity travelers is 155,117 and 107,828, respectively, accounting for 59.0% and 41.0% of the total number of intercity travelers, respectively in May 2021, which indicate that the proportion of male and female travelers has hardly changed. However, it is shown that C (Gender) [T.2] is positive when Y = 0.2, Y = 0.4, Y = 0.6, Y = 0.8, and Y = 1 in Table 11, while C (Gender) [T.2] is negative in the model of 2019. Compare to preferring choosing the intercity travel mode of the private car–HSR–private car in 2019, women start to try other travel modes.
- The influence of whether the travel day is a weekday or not is largely consistent between May 2019 and May 2021. The change is that the predictive effect of weekday travel on the railway–HSR–private car chain changes from a negative effect to a positive effect. Meanwhile, its predictive effect on private car–HSR–railway and public transportation–HSR–private car increases, suggesting that travelers prefer a private car with less exposure risk of COVID-19 for weekday travel. There is previous study found that more people prefer to use private cars and bikes instead of public transportation modes during the pandemic [83]. Before the COVID-19 outbreak, car use was more limited because of its high environmental and social costs, including pollution, obesity, energy consumption, and other negative externalities [84]. In China, highway tolls were suspended from 17 February 2020 to 6 May 2020 by the Ministry of Transport of the People’s Republic of China, to encourage people to travel by car during the pandemic [85,86].
- The prediction of age characteristics on the trip chain changes mainly in the public transportation-HSR-railway trip chain, and the predictive effects of the age group above 39 years old for this trip chain all change from negative to positive during the post-COVID-19 phase.
- The effect of travel distance within the origin city in travel mode is similar in 2019 and 2021 with a slight difference in that the positive effect diminishes and the negative effect increases in the travel mode of public transportation –HSR–private car and public transportation–HSR–railway. The result is consistent with the previous study that for longer distances, people shifted from public transport to private car [87]. It is probably because COVID-19 leads to travelers preferring private cars instead of public transportation due to the threats of infection. This finding can also be seen in the effect of traveler’s waiting time at the starting city transportation hub, compared to 2019, the positive effect of waiting time on public transportation (Y = 0.8, Y = 1) predicting relative to other modes of travel decreases in the year 21. There has not been a significant difference in the influence of travel distance and waiting time in the process of traveling within destination cities between 2019 and 2021.
- Compared with the period pre-outbreak of the pandemic, intercity travelers who travel from home have an increasingly positive effect on the travel modes of the private car during the post-COVID-19 phase. Individuals also tend to temporarily restrict travel for daily social and economic activities or use relatively low-risk modes of transportation to avoid contracting infectious diseases which is consistent with previous studies [88,89].

#### 5.3.3. Comparison Analysis of Travel Behavior between 2019 and 2021 from Table 10, Table 11 and Table 12

- The travel behavior of intercity high-speed railway travelers during the COVID-19 pandemic has changed significantly by analyzing the model parameters of 2019 and 2021. In the odds ratios results of the Multinomial Logit model as shown in Table 12, the parameters of white, red, and green, respectively, represent odds ratios equal to 1, odds ratios are less than 1 and odds ratios are greater than 1. It is obvious that the odds ratios of the same independent variables and dependent variables vary between the years 2019 and 2021. Studies found that COVID-19 reduces intercity travel directly and indirectly by influencing industry development and transport connectivity [90].
- In the aspect of travel mode choice, travelers tend to choose the trip chain of public transportation–HSR–public transportation in 2019 while travelers tend to choose the trip chain of private car–HSR–public transportation in 2021. The spread of COVID-19 has decreased the willingness to choose public transport where travelers are more likely to be infected due to intensive passenger flow. On weekdays, the mode choice of the private car is significantly increasing in 2021.
- Depending on the variable of C (Activity_type), it is found that the willingness to use a private car for trips on the home-based side is increasing with the value of odds ratios increasing by about 20%.
- However, the odds ratios value of variable C (Travel_Wt_O) decreased by about 40% in the groups of railway–HSR–private car and public transportation during the post-COVID-19 phase, which indicates that the travel volume transferred from public transportation to private car.
- Compared to the period pre-outbreak of the pandemic, intercity High-speed railway travelers are more willing to travel by private car, which has less risk, during the post-COVID-19 phase [91]. The nature of risk aversion encourages people to use private vehicles instead of public transportation. Interestingly, however, this characteristic was not reflected by gender and age, as female travelers were less likely to use a private car to reach transportation hubs in 2021, which is consistent with the finding that a higher proportion of male travelers transferred to the private car than female travelers after the outbreak in some studies.

## 6. Conclusions

The transportation environment can reflect the status of urban vitality and economic development, whereas the analysis of travel behavior adjustments under COVID-19 and the corresponding influencing factors can help to deepen the understanding of how the pandemic has affected the daily life of individuals, thus helping to analyze the far-reaching influence of the pandemic on public health and society. Meanwhile, analyzing the impact of the pandemic on the transportation system and individual behavior can provide support for public health management and travel environment improvement during the post-COVID-19 phase.

Based on smartphone data, a novel method to generate Spatio-temporal trip chains of intercity high-speed railway travelers, which includes identification of intercity travelers, extraction of travel features within the city, and generation of intercity trip chains is first proposed in this study. Next, the studentized residual method is used to test the validity of the generated trip chains. By the combination of the two methods, a total of 574,221 pieces of trip chain are obtained. Then, a Multinomial Logit model is employed to explore the changes in the travel behavior of intercity High-speed railway travelers during the COVID-19 pandemic from the perspective of a trip chain with A case study of Beijing–Tianjin–Hebei urban agglomeration, China. This study finds a series of changes in travel behavior from the individual perspective between the period pre-outbreak of COVID-19 and the post-COVID-19 phase. These methods and findings can help us understand travel behavior from the individual perspective and provide critical support for the formulation of public health policy and the improvement of the transportation environment during the post-COVID-19 phase.

The contributions of the present study are threefold: (1) The method of generating intercity trip trains possesses excellent applicability which can be directly used in other urban agglomerations. (2) It includes both individual attributes and travel characteristics and explicitly examines the changes in intercity travel behavior. Most previous studies largely focused on changing travel patterns and travel frequency during COVID-19. (3) The findings proposed in this study are expected to guide public health management and travel environment improvement under the situation of normalized COVID-19 prevention and safety control.

There are also some limitations in this study. First, due to the limitation of the data, this study can only analyze and compare the changes in travel behavior between May 2019 and May 2021, while the characteristics of travel behavior are different in the other 11 months. More data is warranted to further compare the results of this study. On the one hand, we plan to purchase more data by applying for funding in the future. On the other hand, we will actively seek cooperation with the communication operator to jointly study this issue. Second, we explored the changes in the travel behavior of intercity high-speed railway travelers in the context of the whole Beijing–Tianjin–Hebei urban agglomeration. In the future, we can further compare the changes in travel behavior on an urban scale. Meanwhile, we can also compare the travel behavior in Beijing–Tianjin–Hebei urban agglomeration with other urban agglomeration, such as China’s Greater Bay Area, the Yangtze River Delta urban agglomeration, and so on.

## Figures and Tables

**Table 1:** The data size.

	May 2019				May 2021			
Region	Number of Users (Million)	%	Data Volume (Billion)	%	Number of Users (Million)	%	Data Volume (Billion)	%
Beijing	11.98	44.2	19.32	48.0	12.26	42.5	26.70	48.6
Tianjin	7.17	26.4	13.73	34.1	7.94	27.6	17.38	31.7
Shijiazhuang	4.79	17.7	3.97	9.9	4.93	17.1	6.32	11.5
Handan	3.17	11.7	3.23	8.0	3.69	12.8	4.49	8.2
Total	27.12	100.0	40.26	100.0	28.81	100.0	54.90	100.0

**Table 2:** Variable Discretization.

Age		Distance (km)		Time (min)	
Initial	Discretization	Initial	Discretization	Initial	Discretization
[16–18]	A1	[0–5]	D1	[0–5]	T1
[19–24]	A2	[6–10]	D2	[6–10]	T2
[25–29]	A3	[11–15]	D3	[11–15]	T3
[30–34]	A4	[16–20]	D4	[16–20]	T4
[35–39]	A5	[21–25]	D5	[21–25]	T5
[40–44]	A6	[26–30]	D6	[26–30]	T6
[45–49]	A7	[31–35]	D7	[31–35]	T7
[50–54]	A8	[36–40]	D8	[36–40]	T8
[55–59]	A9	[41–45]	D9	[41–45]	T9
[60–64]	A10	[46–50]	D10	[46–50]	T10
[65–69]	A11	[51–55]	D11	[51–55]	T11
[70–74]	A12	[56–60]	D12	[56–60]	T12
[75–79]	A13	[61–66]	D13	[61–66]	T13
[80–84]	A14	[66–70]	D14	[66–70]	T14
[85+]	A15	[71–75]	D15	[71–75]	T15
UNKNOWN	A16	…	…	…	…

‘…’ represents that the following contents are omitted due to space limitation.

**Table 3:** Data description.

NO	Variables	Type	Description	Information Fields
1	UID	string	ID of the user	Eighteen coded digits e.g.: 7368173496636100000
2	Gender	string	The sex of the user	1: Male
				2: Female
				3: unknown
3	Age	string	The age of the user	The age groups are shown in Table 2
4	Arpu	double	The telecoms charge of user	Take 50 yuan as the interval e.g.:
				1: 0–50
				2: 50–100
				Therefore, on
5	Area	string	The attribution of the user	Area code e.g.: V0110000
6	Brand	string	The mobile phone brand of the user	Mobile phone brand e.g.: Apple
7	Stime	timestamp	Start time of travel	Specific time e.g.: 2021/5/4 8:48:57
8	Etime	timestamp	End time of travel	Specific time e.g.: 2021/5/4 9:09:34
9	Mode	smallint	Mode of transportation	1: Private car
				2: Railway
				3: Airplane
				4: public transportation
				0: Others
10	Time	bigint	Travel time	The time groups are shown in Table 2
11	Distance	bigint	Travel distance	The distance groups are shown in Table 2
12	Ptype	int	Origin or destination type	1: Residence
				2: Work
				0: Visit
13	City	string	The city where the user is located	e.g.:
				011
				012
14	Lat	double	Weighted centroid latitude	e.g.: 39.95964
15	Lon	double	Weighted centroid longitude	e.g.: 116.0975

**Table 4:** The results of the average trip frequency under different thresholds.

Time Threshold/Min	MinR/Meter		
	300	500	700
10	3.17	3.03	2.91
20	2.94	2.92	2.68
30	2.80	2.73	2.57

**Table 5:** Identification of abnormal values based on the variables of destination travel distance and time (May 2019).

Gender	Wd	Age	OTM	OTD	OTT	DTM	DTD	DTT	Studentized Residuals
1	1	7	1	8	7	2	11	212	31.26656
1	1	7	1	0	0	2	11	212	31.26656
1	1	7	1	8	7	2	11	212	31.26656
1	1	7	1	0	0	2	11	212	31.26656
1	1	7	1	1	1	1	1	196	29.68369
1	1	8	1	3	3	2	3	164	24.65408
1	1	6	1	3	4	1	2	162	24.43505
3	0	16	2	10	11	4	4	157	23.50619
1	1	7	4	2	1	2	19	160	22.69109
1	1	7	4	2	1	2	19	160	22.69109
1	1	5	1	0	0	4	4	146	21.83551
…	…	…	…	…	…	…	…	…	…

Wd denotes character of the day. OTM denotes the travel mode from origin to hub A. OTD denotes the travel distance from origin to hub A. OTT denotes the travel time from origin to hub A. DTM denotes the travel mode from hub B to destination. DTD denotes the travel distance from hub B to destination. DTT denotes the travel time from hub B to destination. The last row represents that the following abnormal values are omitted due to space limitation which is the same in Table 6, Table 7 and Table 8.

**Table 6:** Identification of abnormal value based on the variables of destination travel distance and time (May 2021).

Gender	Wd	Age	OTM	OTD	OTT	DTM	DTD	DTT	Studentized Residuals
1	0	10	1	0	0	2	4	163	24.36926
1	0	11	1	0	0	2	4	163	24.36926
2	0	11	1	2	2	1	1	149	22.47561
1	1	7	1	0	0	2	0	143	21.64260
1	1	6	1	0	0	2	0	143	21.64260
1	1	6	2	16	18	1	61	173	21.60481
2	0	7	1	13	14	2	45	165	21.59465
1	0	7	1	1	1	1	3	142	21.26605
1	1	11	2	2	13	1	8	144	21.19340
2	1	6	1	24	16	4	3	140	20.96335
2	1	6	1	24	16	4	3	140	20.96335
…	…	…	…	…	…	…	…	…	…

**Table 7:** Identification of abnormal values based on the variables of origin travel distance and time (May 2019).

Gender	Wd	Age	OTM	OTD	OTT	DTM	DTD	DTT	Studentized Residuals
2	1	6	4	13	252	1	0	0	36.42732
2	1	7	4	13	252	1	0	0	36.42732
2	0	6	1	5	195	1	0	4	29.27448
1	1	6	4	8	198	1	0	0	29.09692
1	1	8	1	2	182	2	8	9	27.89576
1	1	7	1	2	182	2	8	9	27.89576
1	1	8	4	1	180	1	0	0	27.79935
1	1	6	1	2	180	1	0	0	27.58449
1	1	7	1	2	180	1	0	0	27.58449
1	1	7	1	2	180	1	0	0	27.58449
1	1	7	1	2	180	1	0	0	27.58449
…	…	…	…	…	…	…	…	…	…

**Table 8:** Identification of abnormal value based on the variables of origin travel distance and time (May 2021).

Gender	Wd	Age	OTM	OTD	OTT	DTM	DTD	DTT	Studentized Residuals
1	0	7	4	4	250	2	7	7	35.12926
1	0	6	4	4	250	2	7	7	35.12926
1	0	7	4	3	231	1	1	0	32.55799
1	0	7	4	3	231	1	1	0	32.55799
1	0	6	4	3	231	1	1	0	32.55799
1	0	8	4	2	229	2	5	6	32.41404
1	0	8	4	2	229	2	5	6	32.41404
1	1	7	4	1	228	4	1	12	32.41288
1	1	7	4	1	228	4	1	12	32.41288
1	0	9	1	0	222	2	1	8	31.69780
2	1	10	4	0	219	1	9	15	31.26945
…	…	…	…	…	…	…	…	…	…

**Table 9:** The independent variables of the model.

NO	Variables	Description	Information Fields
1	Gen	The sex of the user	1: Male
			2: Female
2	Wd	The character of the day	0: Weekend
			1: weekday
3	Age	The age of the user	The groups are shown in Table 2
4	Travel_DS_O	The travel distance from the origin to hub A	The groups are shown in Table 2
5	Travel_Wt_O	The waiting time in hub A	The groups are shown in Table 2
6	Travel_Wt_D	The waiting time in hub B	The groups are shown in Table 2
7	Travel_DS_D	The travel distance from hub B to the destination	The groups are shown in Table 2
8	Activity_type	The origin type and destination type	1: Residence
			2: Work
			0: Visit

**Table 10:** Simulation results of multinomial logit model (May 2019).

	Y = 0.2		Y = 0.4		Y = 0.6		Y = 0.8		Y = 1	
	Coeff.	Sig.	Coeff.	Sig.	Coeff.	Sig.	Coeff.	Sig.	Coeff.	Sig.
Intercept	−4.5889	0.000	0.6544	0.000	−2.1786	0.000	−0.2456	0.032	−4.6162	0.000
C(Gen)[T.2]	−0.0222	0.311	−0.0097	0.720	−0.0378	0.257	−0.0393	0.037	−0.0738	0.000
C(Wd)[T.1]	0.0344	0.114	−0.1395	0.000	−0.0002	0.994	−0.1720	0.000	0.0039	0.0851
C(Age)[T.2]	0.2396	0.030	−0.1799	0.105	−0.0153	0.912	0.0341	0.681	0.2029	0.043
C(Age)[T.3]	0.1470	0.187	−0.3970	0.000	0.0319	0.821	−0.2641	0.002	−0.0623	0.539
C(Age)[T.4]	0.0863	0.447	−0.4765	0.000	−0.0704	0.628	−0.2980	0.001	−0.2093	0.044
C(Travel_DS_O)[T.2]	−0.2162	0.000	0.0549	0.423	2.1383	0.000	0.6987	0.000	0.6386	0.000
C(Travel_DS_O)[T.3]	−0.0640	0.557	0.1282	0.298	3.0395	0.000	0.0136	0.857	−0.2819	0.005
C(Travel_DS_O)[T.4]	−0.2598	0.000	0.3349	0.000	−1.1706	0.000	−1.7922	0.000	−1.7418	0.000
C(Travel_Wt_O)[T.2]	0.6786	0.000	0.6002	0.000	0.9021	0.000	0.1253	0.003	1.0836	0.000
C(Travel_Wt_O)[T.3]	0.6082	0.000	0.4456	0.000	0.7887	0.000	0.1435	0.001	1.0195	0.000
C(Travel_Wt_O)[T.4]	0.3656	0.000	0.3644	0.000	0.9570	0.000	−0.0884	0.034	0.5390	0.000
C(Travel_Wt_D)[T.2]	1.4263	0.000	−1.6464	0.000	0.0738	0.497	0.4816	0.000	1.8115	0.000
C(Travel_Wt_D)[T.3]	1.3904	0.000	−1.6642	0.000	−0.1021	0.363	0.4307	0.000	1.8635	0.000
C(Travel_Wt_D)[T.4]	2.2573	0.000	−1.7769	0.000	0.0424	0.692	0.5151	0.000	2.6710	0.000
C(Travel_DS_D)[T.2]	2.2082	0.000	−0.2867	0.000	−0.0532	0.374	−0.0609	0.095	2.0805	0.000
C(Travel_DS_D)[T.3]	3.1017	0.000	−1.0039	0.000	−0.0624	0.586	0.2888	0.000	3.2953	0.000
C(Travel_DS_D)[T.4]	−0.2432	0.000	−1.6815	0.000	−0.2046	0.000	0.4804	0.000	0.2049	0.000
C(Activity_type)[T.10]	1.2316	0.000	0.0277	0.396	−0.7840	0.000	0.1020	0.000	1.1270	0.000
C(Activity_type)[T.12]	1.0072	0.000	0.4400	0.000	−0.6911	0.000	0.2243	0.000	0.8542	0.000
C(Activity_type)[T.21]	−0.0650	0.295	0.0475	0.303	−0.3561	0.000	0.1920	0.000	0.0864	0.106

**Table 11:** Simulation results of multinomial logit model (May 2021).

	Y = 0.2		Y = 0.4		Y = 0.6		Y = 0.8		Y = 1	
	Coeff.	Sig.	Coeff.	Sig.	Coeff.	Sig.	Coeff.	Sig.	Coeff.	Sig.
Intercept	−4.5889	0.000	0.6544	0.000	−2.1786	0.000	−0.2456	0.032	−4.6162	0.000
C(Gen)[T.2]	−0.0222	0.311	−0.0097	0.720	−0.0378	0.257	−0.0393	0.037	−0.0738	0.000
C(Wd)[T.1]	0.0344	0.114	−0.1395	0.000	−0.0002	0.994	−0.1720	0.000	0.0039	0.0851
C(Age)[T.2]	0.2396	0.030	−0.1799	0.105	−0.0153	0.912	0.0341	0.681	0.2029	0.043
C(Age)[T.3]	0.1470	0.187	−0.3970	0.000	0.0319	0.821	−0.2641	0.002	−0.0623	0.539
C(Age)[T.4]	0.0863	0.447	−0.4765	0.000	−0.0704	0.628	−0.2980	0.001	−0.2093	0.044
C(Travel_DS_O)[T.2]	−0.2162	0.000	0.0549	0.423	2.1383	0.000	0.6987	0.000	0.6386	0.000
C(Travel_DS_O)[T.3]	−0.0640	0.557	0.1282	0.298	3.0395	0.000	0.0136	0.857	−0.2819	0.005
C(Travel_DS_O)[T.4]	−0.2598	0.000	0.3349	0.000	−1.1706	0.000	−1.7922	0.000	−1.7418	0.000
C(Travel_Wt_O)[T.2]	0.6786	0.000	0.6002	0.000	0.9021	0.000	0.1253	0.003	1.0836	0.000
C(Travel_Wt_O)[T.3]	0.6082	0.000	0.4456	0.000	0.7887	0.000	0.1435	0.001	1.0195	0.000
C(Travel_Wt_O)[T.4]	0.3656	0.000	0.3644	0.000	0.9570	0.000	−0.0884	0.034	0.5390	0.000
C(Travel_Wt_D)[T.2]	1.4263	0.000	−1.6464	0.000	0.0738	0.497	0.4816	0.000	1.8115	0.000
C(Travel_Wt_D)[T.3]	1.3904	0.000	−1.6642	0.000	−0.1021	0.363	0.4307	0.000	1.8635	0.000
C(Travel_Wt_D)[T.4]	2.2573	0.000	−1.7769	0.000	0.0424	0.692	0.5151	0.000	2.6710	0.000
C(Travel_DS_D)[T.2]	2.2082	0.000	−0.2867	0.000	−0.0532	0.374	−0.0609	0.095	2.0805	0.000
C(Travel_DS_D)[T.3]	3.1017	0.000	−1.0039	0.000	−0.0624	0.586	0.2888	0.000	3.2953	0.000
C(Travel_DS_D)[T.4]	−0.2432	0.000	−1.6815	0.000	−0.2046	0.000	0.4804	0.000	0.2049	0.000
C(Activity_type)[T.10]	1.2316	0.000	0.0277	0.396	−0.7840	0.000	0.1020	0.000	1.1270	0.000
C(Activity_type)[T.12]	1.0072	0.000	0.4400	0.000	−0.6911	0.000	0.2243	0.000	0.8542	0.000
C(Activity_type)[T.21]	−0.0650	0.295	0.0475	0.303	−0.3561	0.000	0.1920	0.000	0.0864	0.106

**Table 12:** Odds Ratios of multinomial logit model.

	May 2019					May 2021				
	Y = 0.2	Y = 0.4	Y = 0.6	Y = 0.8	Y = 1	Y = 0.2	Y = 0.4	Y = 0.6	Y = 0.8	Y = 1
Intercept	0.010164	1.924057	0.113195	0.78221	0.00989	0.014673	0.692959	0.142148	1.693416	0.014864
C(gen)[T.2]	0.978051	0.990327	0.962933	0.961461	0.928881	1.073599	1.143503	1.13668	1.078757	1.006918
C(Wd)[T.1]	1.034981	0.869772	0.999764	0.841938	1.003894	1.313115	0.89642	1.078122	0.839439	1.222483
C(Age)[T.2]	1.270777	0.835363	0.984828	1.034653	1.224895	1.155085	0.914852	0.948327	0.991449	1.454457
C(Age)[T.3]	1.158389	0.672329	1.032439	0.76787	0.939572	1.195872	0.841337	0.985366	0.864334	1.319184
C(Age)[T.4]	1.090116	0.620936	0.932052	0.742273	0.81113	1.163149	0.749763	0.949911	0.901371	1.310159
C(Travel_DS_O)[T.2]	0.805605	1.056385	8.485217	2.009388	1.893753	0.911022	1.18897	8.342486	1.926511	1.929149
C(Travel_DS_O)[T.3]	0.938046	1.136811	20.894082	1.013696	0.754336	0.867306	1.117901	23.505125	0.522586	0.447203
C(Travel_DS_O)[T.4]	0.771203	1.397751	0.31019	0.16659	0.175203	0.852874	1.283957	0.338453	0.173982	0.194905
C(Travel_Wt_O)[T.2]	1.971196	1.822432	2.464805	1.133472	2.955228	1.579472	1.075008	2.108464	0.768869	1.759134
C(Travel_Wt_O)[T.3]	1.837208	1.561375	2.200459	1.154282	2.771823	1.496685	1.115643	1.841175	0.744692	1.337071
C(Travel_Wt_O)[T.4]	1.441325	1.439629	2.603854	0.915396	1.714319	1.337739	1.088986	2.3304	0.571691	0.984857
C(Travel_Wt_D)[T.2]	4.163081	0.192747	1.076551	1.618588	6.119854	3.434241	0.41128	0.974324	0.80034	4.076087
C(Travel_Wt_D)[T.3]	4.016634	0.189349	0.90297	1.538404	6.446181	3.117275	0.420494	1.104252	0.970186	5.318753
C(Travel_Wt_D)[T.4]	9.556902	0.169156	1.043265	1.673798	14.454672	8.277234	0.43898	1.113555	1.075391	14.769172
C(Travel_DS_D)[T.2]	9.098959	0.750763	0.948166	0.940882	8.008254	10.687326	1.554117	0.841836	1.149816	8.737443
C(Travel_DS_D)[T.3]	22.235454	0.366448	0.939494	1.334865	26.986254	24.084075	1.080882	0.877746	1.637618	33.022645
C(Travel_DS_D)[T.4]	0.78414	0.186101	0.81496	1.616682	1.227379	0.725077	0.169348	0.762655	1.579705	1.029895
C(Activity_type)[T.10]	3.426819	1.028073	0.456573	1.107426	3.086265	3.71201	1.240926	0.443314	0.902938	2.446463
C(Activity_type)[T.12]	2.737863	1.552745	0.501	1.251496	2.349454	4.222796	1.343585	0.413599	0.892905	2.667548
C(Activity_type)[T.21]	0.937032	1.048676	0.700391	1.211628	1.090228	0.930034	0.835564	0.668306	1.216374	0.847833

The parameter with white color indicates OR = 1, the parameter with red color indicates OR < 1, and the parameter with green color indicates OR > 1.

## Data Availability

Data sharing not applicable.
